# High-Quality Image Compression Algorithm Design Based on Unsupervised Learning

**DOI:** 10.3390/s24206503

**Published:** 2024-10-10

**Authors:** Shuo Han, Bo Mo, Jie Zhao, Junwei Xu, Shizun Sun, Bo Jin

**Affiliations:** 1 School of Aerospace Engineering, Beijing Institute of Technology, Beijing100081, China; mobo@bit.edu.cn (B.M.); zhaojie@bit.edu.cn (J.Z.); 3120220104@bit.edu.cn (J.X.); 3220225009@bit.edu.cn (S.S.); 2 Chongqing Chang’an Wang Jiang Industry Group Co., Ltd., Chongqing 400023, China; jinbo@bit.edu.cn

**Keywords:** high-quality image compression, content-weighted autoencoder, compression ratio, multi-scale discriminator, unsupervised learning

## Abstract

Increasingly massive image data is restricted by conditions such as information transmission and reconstruction, and it is increasingly difficult to meet the requirements of speed and integrity in the information age. To solve the urgent problems faced by massive image data in information transmission, this paper proposes a high-quality image compression algorithm based on unsupervised learning. Among them, a content-weighted autoencoder network is proposed to achieve image compression coding on the basis of a smaller bit rate to solve the entropy rate optimization problem. Binary quantizers are used for coding quantization, and importance maps are used to achieve better bit allocation. The compression rate is further controlled and optimized. A multi-scale discriminator suitable for the generative adversarial network image compression framework is designed to solve the problem that the generated compressed image is prone to blurring and distortion. Finally, through training with different weights, the distortion of each scale is minimized, so that the image compression can achieve a higher quality compression and reconstruction effect. The experimental results show that the algorithm model can save the details of the image and greatly compress the memory of the image. Its advantage is that it can expand and compress a large number of images quickly and efficiently and realize the efficient processing of image compression.

## 1. Introduction

In the current era of intelligence, the amount of image information on the Internet is showing an exponential growth trend. The information in the network transmission and storage must satisfy two requirements: first, it must be fast and timely without delay; second, it must have low data loss and maintain the integrity of the information content [1,2,3]. Timely transmission of information is one of the important needs of today, and images contain more abundant and specific content and occupy a large proportion of Internet information [4,5]. Faced with the explosive development of information interaction, image information processing has become increasingly cumbersome. Under the conditions of limited network bandwidth and memory resources [6,7], the requirements for image data compression quality and efficiency are becoming increasingly high [8,9]. On the other hand, the more efficient the compression performance, in turn, can greatly promote the development of network transmission capabilities.

Image compression is essentially data compression, and its purpose is to store image data in less space [10,11], thereby improving the transmission efficiency of images in the network and ensuring that the quality of compressed images is not reduced [12,13]. The existing compression methods can achieve good compression of images, but they cannot achieve both compression efficiency and compression quality when compressing a large number of images at the same time [14]. In order to better solve this problem, images can be compressed in a shorter time and with a better compression rate while ensuring higher compression quality. Therefore, this paper proposes a high-quality image compression algorithm based on unsupervised learning, in which a content-weighted autoencoder, an importance map, and a binary quantizer are specially designed. At the same time, a multi-scale discriminator is designed to determine whether the generated compressed image meets the standard, as well as a composite loss function to improve the compression quality and efficiency. Finally, the network training process is redesigned for the compression algorithm to optimize the algorithm network and improve the work efficiency. The corresponding verification experiments of the algorithm and other mainstream algorithms are carried out on the open datasets Kodark24, Cityscapes, and Urban100. The results show that the compressed images generated by the proposed compression algorithm have a better compression ratio, smaller memory usage, more complete detail information, and higher efficiency.

The main work of this study is as follows:

(1) In order to preserve more detailed information of the compressed image and improve the quality of the compressed image generation, a new content-weighted autoencoder, a new importance map, and a binary quantizer are designed. In order to make a more comprehensive judgment on the authenticity and global consistency of the generated compressed image, a multi-scale discriminator is designed.
(2) In order to improve the efficiency and quality of the compressed image, a composite loss function of the composite algorithm model is designed.
(3) Verification experiments are carried out on multiple open datasets. The results show that the proposed algorithm has a better compression ratio and higher efficiency than the traditional algorithm and other advanced algorithms.

## 2. Related Work

Image compression algorithms can be divided into lossless compression methods and lossy compression methods according to whether the image information is lost. Lossless compression [15,16] is commonly used in the fields of medical images, fingerprint images, remote sensing images, etc. Representative algorithms include: (1) Huffman coding; (2) Run-length coding; and (3) Arithmetic coding. Image lossy coding is a compression method with a very high compression ratio [17,18,19]. It is most commonly used in people’s production and life, such as network images, streaming media, videos, and other scenes. Representative algorithms include: (1) Predictive coding; (2) Fractal coding; (3) Subband coding; (4) JPEG; and (5) JPEG2000.

**Figure 1:**
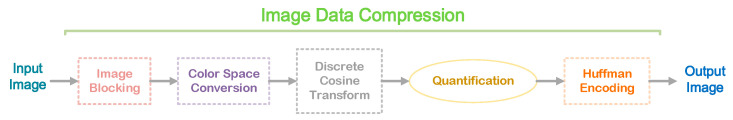
JPEG image compression principal process.

JPEG is often used for digital image compression processing. Its lossy compression method based on discrete cosine transform is simple and efficient, but easy to distort. The principle flow of JPEG image compression is shown in Figure 1.

**Figure 2:**
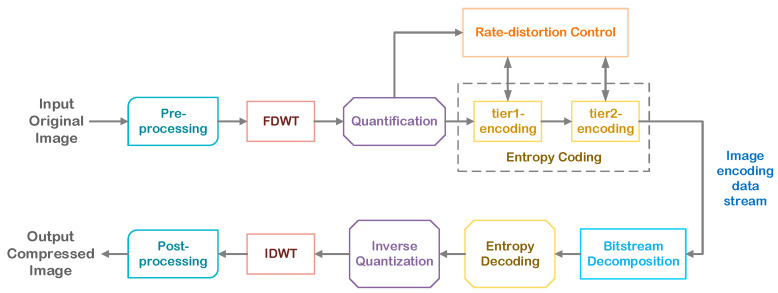
JPEG2000 image compression encoding and decoding system process.

In addition, the upgraded version of JPEG, JPEG2000, is based on the discrete wavelet (DWT) multi-channel analytical image compression coding method with a higher compression ratio. Through calculation, the image signal can be divided more finely, and the high frequency and low frequency are both good. The processing effect is good, but the disadvantage is that it takes a long time and cannot process a large amount of image data in a short time. The JPEG2000 image compression coding and decoding system process is shown in Figure 2.

The encoding process of JPEG2000 requires preprocessing of the image first, in which slicing is for better slicing processing. Slicing does not require the shape and size, and each small block is encoded separately. Another part of the preprocessing canvas is to set the area of the image. Then FDWT (wavelet transform) is performed. After the wavelet is performed, subbands will be generated. Different quantization steps are used for different subbands to obtain quantization results. Then entropy coding is performed. After the entropy coding is completed, several rectangular blocks will be formed. The rectangular blocks divide the area into code blocks. The bit planes of the blocks are encoded from high to low to obtain a bit stream. After encoding all the code blocks, they are put together according to the same bit stream to form a code stream organization. After that, after decoding and quantization, IDWT transformation is performed, and finally the compressed image is output after post-processing.

Different from the traditional image compression algorithm, the image compression algorithm based on deep learning has developed rapidly due to its great potential, high compression ratio, and fast decompression speed [20,21,22,23]. In particular, this type of deep learning image compression algorithm can be deployed on a low-cost hardware platform without restriction, which makes it a hot spot in the study of image compression algorithms.

Zhang et al. [24] proposed a piecewise differential coding method based on structural statistical saturation, aiming to improve the usability of video while ensuring compression integrity. Agustsson et al. [25] proposed a generative compression method by optimizing the rate-distortion-realism trade-off, which can generate detailed and realistic images at low bit rates and avoid the blurry reconstruction produced by traditional rate-distortion optimization models. Bai et al. [26] proposed a unified and powerful deep lossy and residual (DLPR) coding framework suitable for lossless and near-lossless image compression. Jiang et al. [27] proposed a quantization error compensation method that can flexibly combine multiple end-to-end learning image compression techniques. Fu et al. [28] designed an efficient image-coding framework with an asymmetric structure. The encoder uses three-stage multi-scale residual blocks (MSRBs), while the decoder only uses one stage, which reduces the decoding complexity while maintaining good performance.

Wang et al. [29] proposed a new framework for compressed images based on multi-scale dilated convolutional neural networks for compressed sensing (CS) measurement and reconstruction, which directly obtains measurements from the trained full convolutional structure and avoids traditional block effects. Lau et al. [30] developed an SPI system based on block compressed sensing (BCS) and U-Net CNN, which provides an opportunity to pre-train deep learning models for BCS reconstruction of images in various fields. Duan et al. [31] redesigned their latent variable model and simplified quantization and entropy coding in image compression by quantizing the a priori and priori of perception. Al-khassaweneh et al. [32] designed a method based on the Frei-Chen base technique and modified run length encoding (RLE) to compress images. The goal of RLE is to improve the compression factor without adding any distortion. Yang et al. [33] proposed an end-to-end optimized lossy image compression framework, which introduces an additional “content” latent variable that is a condition for the back diffusion process and uses this variable to store information about the image.

The above algorithm designs have good performance in special scenarios, but it still needs to be improved in the compression speed and compression rate, and it is difficult to face the complex and changeable timeliness and other common application scenarios due to the lack of generalization effect. At the same time, the widespread use of end-to-end smart devices urgently requires high-quality image compression algorithms with good performance and smaller memory usage.

## 3. Model Establishment

This paper studies high-quality image compression technology. The compressed images are used for fast transmission of image information and to reduce memory usage. Therefore, high requirements are placed on compression speed and image details. In the past, the image compression algorithm based on an autoencoder was designed [34,35]. Through reasonable allocation of positions, the image compression effect was significantly optimized. However, for some high resolution images, the compression did not achieve good results and still had blurring and distortion problems. The generative adversarial network has excellent image compression performance for high resolution images. Deep learning promotes the development of image compression technology, continuously improves the performance of image compression, and achieves better compression rate and better compression index. Therefore, it is very important to learn to establish a better image compression framework. Therefore, this paper re-designs the content-weighted autoencoder as the basis of image compression and deeply integrates it with the generative adversarial network to form a framework for high-quality image compression so as to achieve the maximum preservation of image information at a faster compression speed and better compression rate. The following first introduces the overall network structure of the high-quality image compression algorithm designed in this paper and then expands the key modules and the loss function used in detail. Finally, the training and use of this algorithm are explained.

### 3.1. Overall Structure Design of Algorithm Network

**Figure 3:**
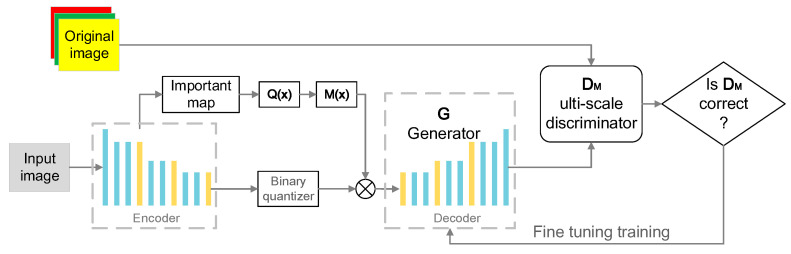
Overall structure design of algorithm network.

The network structure of the high-quality image compression algorithm designed in this paper includes the following main modules: content-weighted autoencoder, in which the decoding part is used as the generator G in the generative adversarial network to realize the function of the generator, and the compressed data output by the autoencoder can be used as the generation condition; importance map, in which Q(x) represents the importance map quantization process and M(x) represents the importance map mask calculation process; binary quantizer, which can set the activation function in the encoder and convert it with the activation function to generate the decoding result during decoding; multi-scale discriminator D_M_ and composite loss function *L*_cos_. The above modules together realize high-quality image compression, and the overall structure design of the algorithm network is shown in Figure 3.

### 3.2. Content-Weighted Autoencoder

The content-weighted autoencoder uses convolution operation to replace the traditional fully connected mode of encoding, which can achieve image compression based on a smaller bit rate and optimize the problem with entropy rate. Its structure includes two parts: encoding and decoding. Among them, the encoding part is a structure composed of a cascade combination of convolutional layers and residual modules, including three convolutional layers and three residual blocks. Each residual block has two convolutional layers and a ReLU function. The residual module is used to improve the anti-noise performance of the encoder, and the encoder designed in this paper does not add a normalization layer to avoid visual artifacts in smooth areas.

**Figure 4:**
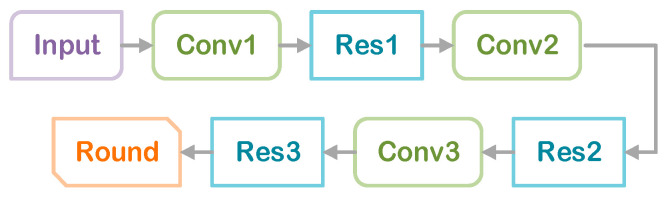
Content-weighted autoencoder encoding process.

In the encoding process, the image is first input into the network, and after convolution through 64 convolution kernels Conv1 with a size of 8 × 8 and a stride of 4, it passes through a residual module Res1. Then it passes through a convolution layer of 128 convolution kernels, Conv2, with a size of 4 × 4 and a stride of 2. After that, it passes through two residual modules, Res2 and Res3, and the feature map is convolved with a 1 × 1 convolution kernel, Conv3. Except for the last layer of the encoder, which uses the Sigmoid activation function, all convolutional layers use ReLU. The encoding process is shown in Figure 4.

The encoding process is to add an activation function to the input signal $x=[x_{1},x_{2},\cdot \cdot \cdot x_{n}]$ and map the data of the input signal to *y*, where *y* is the new data matrix. The mathematical principle is as shown in Formula (1).

(1) y=f(wx+b)

In the formula, *f* is the activation function, *w* is the mapping matrix, and *b* is the bias term of the encoding part.

The decoding part consists of an up-sampling layer and a deconvolution layer. The convolution layer is for feature extraction, and the deconvolution layer is for image reconstruction. Finally, through continuous iteration, the error between the output and the input is minimized to obtain the optimal autoencoder parameters. The feature extraction in the autoencoder is more efficient, and the parameter weights in the convolutional network are shared by neurons, so that the network complexity is easy to train the algorithm model, and the reconstruction quality of the compressed image is improved.

The decoding process is to restore the extracted effective features so that the result is close to the input signal *x*. The mathematical principle is as shown in Formula (2).

(2) $$x^{\prime }=f^{\prime }(w^{\prime }y+b^{\prime })$$

In the formula, $f^{\prime }$ is the mapping function, $w^{\prime }$ is the mapping matrix, and $b^{\prime }$ is the bias term of the decoding part.

### 3.3. Importance Map

In the process of image compression, different regions have different compression difficulties, and smoother regions are easier to compress. However, regions with relatively rich textures are the important parts to obtain information [36,37], so bits should be allocated to parts with complex texture structures. In the process of extracting feature maps, different feature maps contain different information. The content-weighted importance maps can achieve better bit allocation and can control and optimize the compression rate.

**Figure 5:**
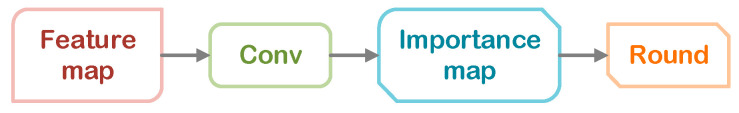
Importance map extraction process.

The importance map is obtained from the input image through learning. The intermediate feature map can be obtained from the residual block of the encoder by processing the image, and then the importance map F(x) is obtained by processing the convolution layer. The importance map extraction process is shown in Figure 5.

In the network, if the input image is *x*, the output of the encoder is $E(x)\in R^{h\times w\times n}$, F(x) is used to represent the importance map of size h×w. When $\frac{l-1}{L}\leq F_{ij}\leq \frac{l}{L}$, encode and store the output information from the first to the $\frac{nl}{L}$ bit. Among them, *L* represents the value of the importance, and $\frac{n}{L}$ represents the bit corresponding to each importance. The importance map is used to realize the allocation of bits. First, the size of the importance map F(x) is recorded as h×w, and the number of feature maps output by the encoder network is recorded as *n*. The importance map is quantized to become an integer less than *n*. Generate an importance feature mask *m* corresponding to B(E(x)), whose size is h×w×n. Here, $f_{ij}$ is recorded as an element in F(x), and the process of obtaining the importance map by quantization is defined as Formula (3).

(3) $$Q(f_{ij})=l-1,\;if\;\frac{l-1}{L}\leq f_{ij}\leq \frac{l}{L},\;l=1,\cdot \cdot \cdot ,L$$

After quantizing the importance map, the importance feature mask *m* is calculated by Formula (4).

(4) $$m_{kij}=\left\{\begin{array}{l} 1\\ n \end{array}\right.if\;k\leq \frac{n}{l}Q(f_{ij})$$

The final encoding result of the input image *x* can be represented by c=M⊗B, where the symbol ⊗ represents element multiplication. In this way, the content-weighted importance map is obtained, which guides the generation of images with clearer textures.

In the process of back propagation, the gradient still needs to be calculated. The feature map is convolved to generate the importance map. The importance feature mask is generated by the valuer, which causes the gradient of most areas to be zero. To calculate the gradient of the mask of the element $p_{ij}$ in the importance map, see Formula (5).

(5) $$m_{kij}=\left\{\begin{array}{l} 1\\ 0 \end{array}\right.if\;\left|\frac{kl}{n}\right|\leq Lp_{ij}$$

### 3.4. Binary Quantizer

After encoding the image, a binary quantizer is used to complete the quantization process. The activation function is the Sigmoid function, which takes values between [0, 1]. After nonlinear transformation, the value output by the encoder should also be between [0, 1]. In forward propagation, the activation value greater than 0.5 can be defined as 1, and the activation value less than 0.5 can be defined as 0, as shown in Formula (6).

(6) $$B(e_{ij})=l,\;if\;\frac{1}{2}<e_{ij}<\frac{l+1}{2},\;l=0,1$$

In back propagation, the gradient is calculated by the chain rule, which will cause the gradient to be almost equal to 0 everywhere during back propagation. In order to solve this problem of gradient descent in back propagation, this paper designs a gradient back propagation function, which is shown in Formula (7).

(7) $$\tilde{B}(x)=x,\;0<x<1$$

### 3.5. Multi-Scale Discriminator

The discriminator is the core of the generative adversarial network. Through adversarial training with the generator, the ability to distinguish the authenticity of the generated image is improved [38,39]. In order to obtain better compression and visual effects, a relatively large receptive field is required. To achieve this goal, a large convolution kernel or a more complex network is required, which will lead to overfitting, so a better convolutional network system needs to be introduced. The multi-scale discriminator can collect feature data at each scale, obtain a better global view and more accurate detail information, and fuse the data at each level through the multi-scale discriminator so that the compressed image generated is as close to the original image as possible.

**Figure 6:**
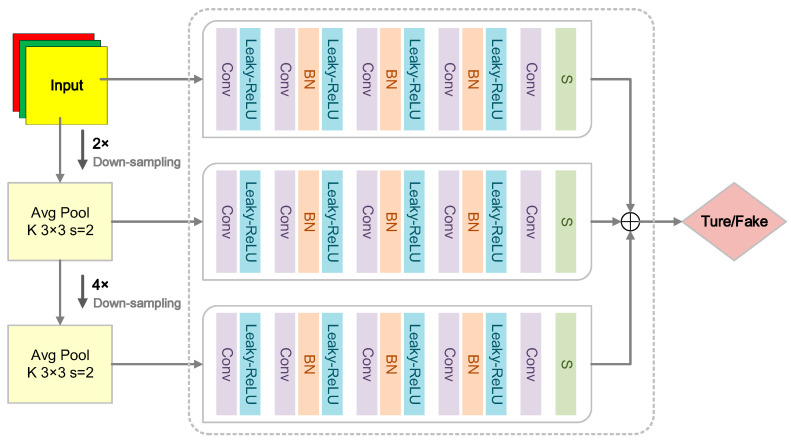
Multi-scale discriminator network structure.

When the image data generated by the content-weighted autoencoder is input into the multi-scale discriminator, the pooling layer will down-sample the input data at different scales to obtain images of three different resolutions and then use three discriminator networks to process the images of the three different scales. Among them, the low-resolution discriminator can obtain a larger field of view when training, and the high-resolution discriminator can minimize image distortion when training, and the generated compressed image texture is clearer. The network structure of the multi-scale discriminator is shown in Figure 6.

The multi-scale discriminator aims to improve its discriminative ability by training the generated compressed images and the original images. As shown in Figure 6, the three discriminator submodules at different scales in the multi-scale discriminator network structure have the same network structure, which consists of two convolutional layers, three convolutional block layers, and a Sigmoid function. Each convolutional block structure consists of a conv, a BN layer, and a Leaky-Relu. The number of convolutional layer operations increases successively. The first layer *n* = 128, the second layer *n* = 256, and the third layer *n* = 512. In the discriminator structure, the convolution kernels in all convolutional networks are 4 × 4, the stride in the first convolutional layer and the convolutional block is 2, and the stride of the last convolutional layer is 1. The specific working principle of the multi-scale discriminator is as follows: 1. Down-sampling processing: First, the image generated by the generator and the original image are subjected to twice and four times down-sampling, respectively, to generate images of three different sizes. The diversity of these scales helps to capture features at different levels. 2. Convolution processing: Then, the images of these three scales are respectively input into the submodules of the discriminator network for convolution processing to extract more detailed feature information. 3. Fusion and judgment: Finally, the multi-scale discriminator adopts parallel add operation fusion for images of various scales, which helps to improve the detection ability of the discriminator network for features of different scales. After fusion, these images are detected and comprehensively judged, and if the output result is ‘True’, it is validly generated; if the output result is ‘Fake’, it is invalidly generated.

### 3.6. Composite Loss Function

The image compression design based on unsupervised learning introduces adversarial networks into the end-to-end framework for generative image compression, so the loss function consists of content-weighted autoencoder loss function, decoder loss function, feature matching loss function, and multi-scale discriminator loss function.

In the process of image data compression and reconstruction by a content-weighted autoencoder, errors will be generated between input data and reconstructed data; that is, data volume will be lost. To achieve better learning and extraction of image data features, it is necessary to constantly adjust the distortion and bit rate of image reconstruction. The rate-distortion function is optimized and used as its loss function, as shown in Formula (8).

(8) $$L_{C}=L_{D}+\alpha L_{R}$$

In the formula, $L_{D}$ represents the rate-distortion loss, α is the weight used to adjust the bit rate, $L_{R}$ and is the rate loss.

The rate-distortion loss is expressed using the *L*_2_ norm square, as shown in Formula (9).

(9) $$L_{D}={\left\|{x^{\prime }}_{n}-x_{n}\right\|}_{2}^{2}$$

In the formula, ${x^{\prime }}_{n}$ represents the reconstructed image, $x_{n}$ represents the input image.

In the network structure of the autoencoder, the rate loss is defined as the entropy of the intermediate feature map, and the encoder hidden space data stored is related to the degree of concentration of the quantized data, so the entropy of the intermediate data is selected to define $L_{R}$, see Formula (10).

(10) $$L_{R}=-E\left[\log_{2}P_{q}\right]$$

In the formula, $P_{q}=\int_{x-\frac{1}{2}}^{x+\frac{1}{2}}P_{d}$, $P_{d}$ represents the probability density function of the original data.

Therefore, the content-weighted autoencoder loss function can be expressed as Formula (11).

(11) $$L_{C}={\left\|{x^{\prime }}_{n}-x_{n}\right\|}_{2}^{2}+\alpha \left\{\left.-E\left[\log_{2}P_{q}\right]\right\}\right.$$

The decoder is a generator in the generative antagonism network, which needs to allocate bits in the process of image compression. The rate-distortion function can optimize the balance between the reconstruction function and the bit rate. The rate-distortion function formula is (12).

(12) $$L_{d}+\beta R=L_{d}+\beta H(\overset{⌢}{w})$$

The loss function of the optimized generator is Formula (13).

(13) $$L_{G}=E_{x∼p_{x}}\left[\lambda R+d(x,\overset{⌢}{x})-\beta lb(D(\overset{⌢}{x},y))\right]$$

In the formula, $d(x,\overset{⌢}{x})$ is the loss, λ and β are weight parameters.

The feature matching loss is represented by MAE (mean absolute error) here, which is less susceptible to outliers than MSE (mean square error), as shown in Formula (14).

(14) $$L_{FM}=E\sum_{i=1}^{L1}\frac{1}{N_{i}}\left[\left\|F_{D}^{i}(x)-F_{D}^{i}(G(z))\right\|\right]$$

The loss function of the multi-scale discriminator is defined in Formula (15).

(15) $$L_{M}=E_{\overset{⌢}{x}∼p_{g}}\left[D(\overset{⌢}{x})\right]-E_{x∼p_{r}}\left[D(x)\right]+\lambda E_{\overset{⌢}{x}∼p_{\overset{⌢}{x}}}\left[{\left({\left\|\nabla_{\overset{⌢}{x}}D(\overset{⌢}{x})\right\|}_{2}-1\right)}^{2}\right]$$

Therefore, the above loss functions together constitute a composite loss function, which can effectively improve the quality and effect of image compression generation from many aspects. The composite loss function is defined as Formula (16).

(16) $$L_{com}=\rho L_{C}+\varphi L_{G}+\phi L_{FM}+\psi L_{M}$$

In the formula, ρ, φ, ϕ and ψ, are weight parameters. According to the experimental platform environment, the weight parameters are continuously adjusted through experiments to achieve the best image compression effect. The weight parameters selected in this paper are 0.5, 0.5, 5, and 3, respectively.

### 3.7. Algorithm Training Process

The specific process of algorithm training is as follows:

Step 1: Use the paired original image and input image as training data, and send the input image to the generator after passing through the encoder, binary quantizer, and importance map calculation to generate a compressed image;

Step 2: Send the generated compressed image and the original image to the multi-scale discriminator D_M_, and the multi-scale discriminator D_M_ will discriminate the two and judge whether the result has achieved the compression effect. If it reaches the standard, it will output the compressed image. If it does not reach the standard, it will continue to return the reconstructed image until a usable compressed image is generated. According to the results, the multi-scale discriminator loss, decoder loss, and content-weighted loss are calculated;

Step 3: Compare the generated compressed image with the original image, and calculate the feature matching loss;

Step 4: Back propagate, according to the losses calculated in steps 2 and 3 above, and update the multi-scale discriminator D_M_ and generator G parameters, respectively;

Step 5: Execute steps 1 to 4, where the input image is given as *x* in the encoder, and the encoder output is obtained by analyzing and transforming the input signal, which is recorded as $E(x)\in R^{h\times w\times n}$. Here h×w is the size, and *n* represents the number of feature maps. The obtained is quantized by the valuer. Based on the characteristics of the binary valuer, the part of the output data greater than 0.5 is marked as 1, and the rest is marked as 0. The feature map in the encoder is extracted, and the network convolution operation is performed separately to obtain the importance map, which is recorded as F(x). Similarly, the quantization operation is carried out on F(x), and the importance mask of the same size is generated after the quantization process as that after E(x) quantization. The obtained importance mask is combined with the binary code generated by the valuer output of the encoder so that the image can better preserve important information, and finally an image compression code is obtained. The decoder is symmetrical with the encoder structure, and the same analysis and transformation are performed to obtain the output result of the decoder and generate a compressed image. When the parameters of D_M_ are updated, the discrimination of effective generation compression can be close to 1, and the discrimination of invalid generation compression can be close to 0, that is, the multi-scale discriminator D_M_ is maximized and optimized. During the training of the generative network, the generator G is connected in series with the multi-scale discriminator D_M_, and the error generated is passed to the generative network. At this time, the parameters of the generative network need to be minimized, that is, the generator G is minimized and optimized. In this process, the generator G and the multi-scale discriminator D_M_ form a dynamic game, and the loop is jumped out after reaching the Nash equilibrium. The generated compressed image is judged to be infinitely close to the real original image.

Step 6: Finally, adjust the parameters of each step according to specific needs, and the algorithm network outputs a usable, high-quality compressed image.

## 4. Verification and Analysis

### 4.1. Experimental Platform

The experimental verification of the algorithm in this paper requires a good experimental platform as a basic condition, mainly including key devices such as CPU, GPU, RAM, and programming framework. The specific parameters are shown in Table 1.

### 4.2. Evaluation Indicators of Compressed Images

Whether the image compression is excellent and whether it can maintain a good compression effect while retaining image information to the greatest extent requires scientific and objective evaluation indicators to compare and analyze the algorithm in this paper with other image compression algorithms. The objective evaluation indicators used in this algorithm include peak signal-to-noise ratio (PSNR), multi-scale structural similarity (MS-SSIM), and compression ratio BPP.

(1) PSNR

PSNR is an important indicator commonly used to measure the quality of image or video generation or compression [40]. The comparison between the compressed image and the original image is generally to compare the pixels in each part of the image. PSNR can intuitively represent the pixel difference between the original image and the reconstructed image. The larger its value, the smaller the image distortion, that is, the better the image quality, as shown in Formula (17).

(17) $$PSNR=20\cdot \log_{10}\left(\frac{MAX(x)}{\sqrt{MSE}}\right)$$

In the formula, MAX(x) represents the maximum value of the pixel grayscale in image *x*. The mean square error (*MSE*) can directly reflect the accuracy loss of the original image and the reconstructed image [41]. The mathematical principle is to take the square average of the difference between the original image and the reconstructed image. The sizes of images *x* and $x^{\prime }$ are both m×n, x(i,j) is the grayscale value of the pixel at position (i,j), and $x^{\prime }(i,j)$ is the gray value of the pixel after compression. The mathematical expression of *MSE* is shown in Formula (18).

(18) $$MSE=\frac{1}{mn}{\sum_{i=0}^{m-1}\sum_{j=0}^{n-1}\left[x(i,j)-x^{\prime }(i,j)\right]}^{2}$$

(2) MS-SSIM

MS-SSIM is an important indicator for evaluating the quality of image compression [42]. It is obtained by multi-scale calculation of SSIM. The purpose is to reduce the impact of different image resolutions on the performance of the compression algorithm. The resolution of the original image is denoted as *Scale*_1_, and the resolution of the image after *M* − 1 iterations is denoted as *Scale*_M_. The contrast measure structure measure in SSIM is calculated at each iterative scale, and the brightness measure is only calculated at the last scale, *Scale*_M_. By synthesizing the results of measurements at different scales, the comprehensive index MS-SSIM is obtained as shown in Formula (19).

(19) $$MS-SSIM(x,x^{\prime })={\left[l(x,x^{\prime })\right]}^{\alpha M}\cdot {\prod_{j=1}^{M}\left[c(x,x^{\prime })\right]}^{\beta j}\cdot {\left[s(x,x^{\prime })\right]}^{\gamma j}$$

In the formula, αM, βj and γj are used to adjust the relative importance of different components. The value range of MS-SSIM is between 0 and 1. The closer the value is to 1, the higher the similarity between the reconstructed image and the original image, and the better the image quality.

(3) BPP

In this paper, BPP is used to measure the size of image compression, and the purpose of compression is that the smaller the BPP, the better [43]. The essence of BPP is the average bit required for each pixel at each position of the image during the compression encoding process. Its mathematical expression is shown in Formula (20).

(20) $$BPP=\frac{pn^{\prime }}{pn}$$

In the formula, the number of original pixels is pn, and the number of compressed pixels is $pn^{\prime }$.

### 4.3. Dataset Preparation and Training Strategy

In order to better train the high-quality image compression algorithm designed in this paper, the ImageNet training dataset is selected. At the same time, in order to objectively verify the superiority of the algorithm in this paper compared with other algorithms, data sets containing different scenes and different topics and data sets with rich texture information are selected as validation data sets. The specific information of the dataset used in the experiment is shown in the following Table 2.

The algorithm model in this paper uses an RTX 3060Ti GPU for network training. The program is written based on the Tensorflow-2.11.0 deep learning framework, and the CPU model is i7-13700F. The batch size is set to 2, and the Adam optimizer is used for training optimization. The network convergence speed is optimized by momentum and adaptive learning rate. The parameters are set to *β*_1_ = 0.5, *β*_2_ = 0.99, and *ε* = 0.5. The initial learning rate is set to 0.0003, which is reduced to 0.0001 after training 100 epochs, and a total of 300 epochs are trained.

### 4.4. Verification of Image Compression Effect on Kodak Dataset

**Figure 7:**
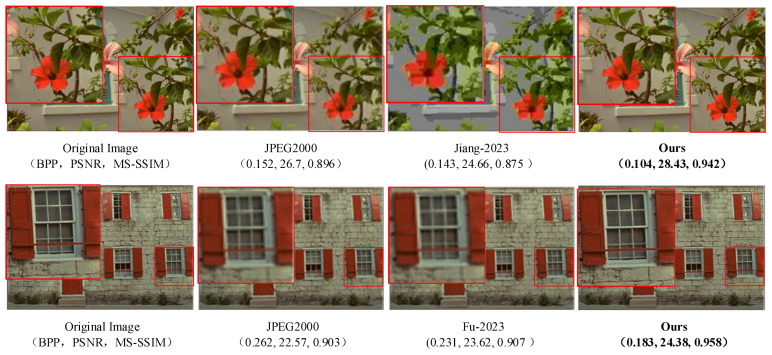
Compression effect of sample images on Kodak24 dataset. Jiang-2023 is the algorithm proposed in reference [27]. Fu-2023 is the algorithm proposed in reference [28].

The specific performance of the high-quality image compression algorithm in this paper needs to be verified on the available test set. The following first selects the images of the Kodak24 dataset to demonstrate the visual compression effect and uses the objective evaluation indicators BPP, PSNR, and MS-SSIM described in Section 4.2 to measure it. Figure 7 shows two sets of comparative experiments, comparing the effects of the compression algorithm in this paper with other advanced compression methods. The sequence from left to right is: uncompressed original image, JPEG2000 compression results, compression results of algorithms in references [27,28], and compression results of the proposed algorithm in this paper. In the compression visualization results, we use red boxes to mark and enlarge the details to compare the compression effects of each algorithm. 

In the first group of experiments, our algorithm is 31.58% lower than the JPEG2000 algorithm in BPP, 6.48% higher in PSNR, and 5.13% higher in MS-SSIM. Compared with the advanced algorithm in the reference [27], our algorithm has reduced the BPP index by 27.27%, significantly improved the PSNR index by 15.29%, and improved the MS-SSIM index by 7.66%.

In the second group of experiments, our algorithm has reduced the BPP index by 30.15%, improved the PSNR index by 8.01%, and improved the MS-SSIM index by 6.09% compared with the JPEG2000 algorithm. Compared with the advanced algorithm in the reference [28], our algorithm has reduced the BPP index by 20.78%, improved the PSNR index by 3.22%, and improved the MS-SSIM index by 5.62%.

The experimental results show that the visualization effect shows that the traditional image compression algorithm JPEG2000 will have visual artifacts such as blur, and other algorithms will have distortion in some samples. The image compression network designed in this paper can better preserve the feature information of the image, and at the same time, the texture details are not seriously lost. The results show that our algorithm has good compression performance and compression effect.

### 4.5. Image Compression Effect Verification on Cityscapes Dataset

**Figure 8:**
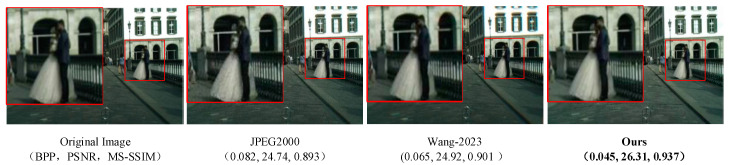
Compression effect of sample images on Cityscapes dataset. Wang-2023 is the algorithm proposed in reference [29].

In order to verify that the high-quality image compression algorithm in this paper has good generalization ability, different test sets are selected for necessary verification. Therefore, images of the Cityscapes dataset are selected to show the visual compression effect, and the above objective evaluation indicators BPP, PSNR, and MS-SSIM are used to measure it. Figure 8 shows the comparison of the compression algorithm in this paper with other mainstream compression methods. The arrangement order from left to right is uncompressed original image, JPEG2000 compression result, compression results of the algorithm in reference [29], and compression results of the proposed algorithm in this paper. In the compression visualization results, we use red boxes to mark and enlarge the details to compare the compression effects of each algorithm.

The objective indicators of this group of experiments show that our algorithm is 45.12% lower than the JPEG2000 algorithm in BPP, 6.35% higher in PSNR, and 4.93% higher in MS-SSIM. Our algorithm is 30.77% lower than the advanced algorithm in reference [29] in BPP, 5.58% higher in PSNR, and 3.99% higher in MS-SSIM.

The experimental results show that from the perspective of visual image effects, JPEG2000 is not much different from the reference [29], and the overall effect is good, but the details still need to be improved, and there are some blurring phenomena on small objects in the distance. The algorithm in this paper has a good processing of detail blur artifacts and can achieve a clearer effect close to the original image, whether it is the shadow in the image or the outline of the person, and the comprehensive performance of objective indicators is far better than other mainstream algorithms.

### 4.6. Verification of Visual Results of Image Compression Reconstruction on Urban100 Dataset

**Figure 9:**
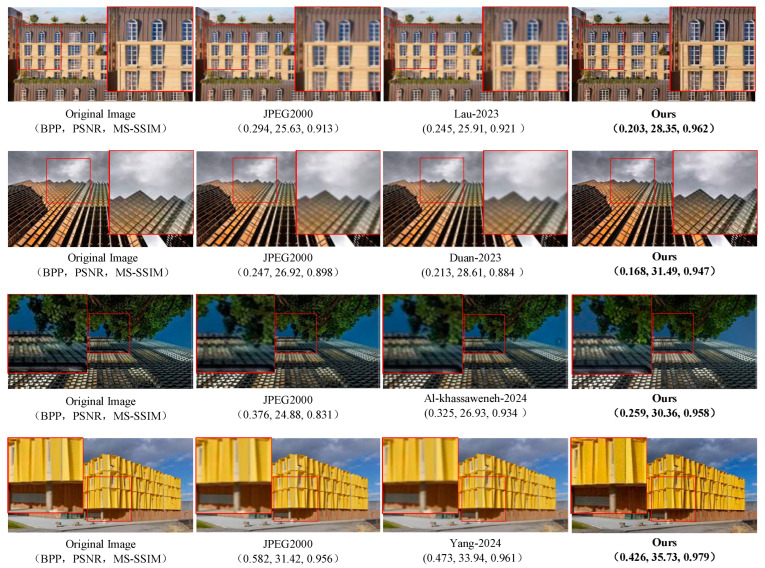
Performance of sample image compression and reconstruction on the Urban100 dataset. Lau-2023 is the algorithm proposed in reference [30]. Duan-2023 is an algorithm proposed in reference [31]. Al-khassaweneh-2024 is the algorithm proposed in reference [32]. Yang-2024 is the algorithm proposed in reference [33].

In order to verify that the image compressed by the proposed algorithm has a good reconstruction effect, the Urban100 data set was tested during the end-to-end training process, and the visualization results of four groups of comparison experiments were obtained, as shown in Figure 9. Its arrangement sequence from left to right is uncompressed original image, JPEG2000 compression reconstruction results, compression reconstruction results of advanced algorithms in references [30,31,32,33], and compression results of the proposed algorithm in this paper. In the compression visualization results, we use red boxes to mark and enlarge the details to compare the compression effects of each algorithm.

In the first group of experiments, our algorithm reduced the BPP index by 30.95% compared with the JPEG2000 algorithm, improved the PSNR index by 10.61%, and improved the MS-SSIM index by 5.36%. Our algorithm reduced the BPP index by 17.14% compared with the advanced algorithm in the reference [30], improved the PSNR index by 9.41%, and improved the MS-SSIM index by 4.45%.

In the second group of experiments, our algorithm reduced the BPP index by 31.98% compared with the JPEG2000 algorithm, improved the PSNR index by 16.97%, and improved the MS-SSIM index by 5.45%. Our algorithm reduced the BPP index by 21.12% compared with the advanced algorithm in the reference [31], improved the PSNR index by 10.06%, and improved the MS-SSIM index by 7.12%.

In the third group of experiments, our algorithm reduces the BPP index by 31.11% compared with the JPEG2000 algorithm, improves the PSNR index by 22.02%, and improves the MS-SSIM index by 15.28%. Our algorithm reduces the BPP index by 20.30% compared with the advanced algorithm in the reference [32], improves the PSNR index by 12.73%, and improves the MS-SSIM index by 2.56%.

In the fourth group of experiments, our algorithm reduces the BPP index by 26.8% compared with the JPEG2000 algorithm, improves the PSNR index by 13.71%, and improves the MS-SSIM index by 2.19%. Our algorithm reduces the BPP index by 9.93% compared with the advanced algorithm in the reference [33], improves the PSNR index by 5.27%, and improves the MS-SSIM index by 1.87%.

Experimental results show that the images compressed and reconstructed by the algorithm in this paper on the Urban100 dataset perfectly restore the visual effects of the original images. For example, in the first group of experiments, the boundary between the building edge and the window of the compressed and reconstructed image by our algorithm was restored very clearly, and there was no splitting phenomenon. In the second group of experiments, our algorithm compressed the clouds and architectural edges in the reconstructed images, and the color and exposure were clearly restored, and there was no fuzzy distortion problem. In the third group of experiments, our algorithm compresses the edge of the leaves and the light reflected by the glass in the reconstructed images and has satisfactory reconstruction effects, and some of the image details prone to blur artifacts, such as overlapping leaves and shaded parts of trees and houses, are well handled. In the fourth group of experiments, the light and dark textures of the yellow buildings in the compressed reconstructed images were well restored by our algorithm, and the edge between the clouds and the buildings did not appear blurred.

### 4.7. Algorithm Performance Evaluation

**Figure 10:**
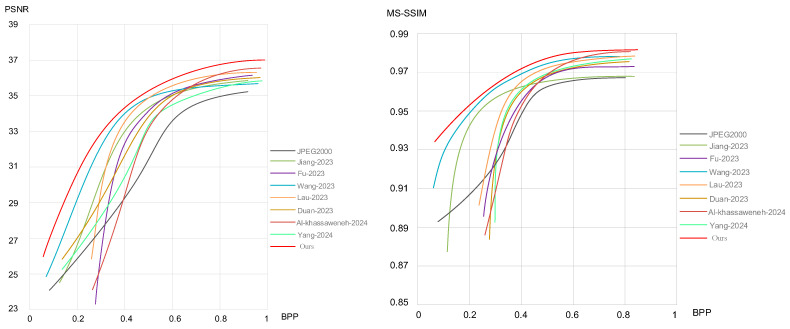
PSNR and MS-SSIM performance test curves of each algorithm. Jiang-2023 is the algorithm proposed in reference [27]. Fu-2023 is the algorithm proposed in reference [28]. Wang-2023 is the algorithm proposed in reference [29]. Lau-2023 is the algorithm proposed in reference [30]. Duan-2023 is an algorithm proposed in reference [31]. Al-khassaweneh-2024 is the algorithm proposed in reference [32]. Yang-2024 is the algorithm proposed in reference [33].

In order to objectively evaluate the performance of the proposed algorithm network, a visual comparison of BPP, PSNR, and MS-SSIM indicators is performed on the above experimental platform with the traditional image compression method JPEG2000 and the advanced algorithms proposed in the references [27,28,29,30,31,32,33]. The above image compression algorithms are all tested on the dataset prepared in this paper. The performance curves of the BPP, PSNR, and MS-SSIM indicators of each image compression algorithm are shown in Figure 10.

As can be seen from Figure 10, when evaluated by the PSNR indicator, our algorithm shows a relatively stable overall performance. When the BPP is low, it has a more obvious advantage over traditional algorithms and other advanced algorithms, and it still maintains a leading position when the BPP is high. When evaluated by the MS-SSIM indicator, our algorithm has a slower growth of MS-SSIM when the BPP is high and has a great advantage over traditional algorithms and other advanced algorithms when the BPP is low. The results show that compared with traditional algorithms and other advanced algorithms, our algorithm has good performance, better image compression and reconstruction quality, and the overall performance has certain advantages.

### 4.8. Ablation Experiment Results Verification

The key functional modules designed in Section 3 above have different effects on image compression performance, so the ablation comparison experiment is designed in this section to test the actual situation of different key modules on compression efficiency. The experiment includes four test groups and one control group. The specific information is as follows: 1. Replace the content-weighted autoencoder with a normal encoder; 2. Replace the importance map network with a normal feature extraction convolutional network; 3. Replace the binary quantizer with a normal quantizer; 4. Replace the multi-scale discriminator with a general discriminator; 5. A complete network of the high-quality image compression algorithm designed in this paper.

**Figure 11:**
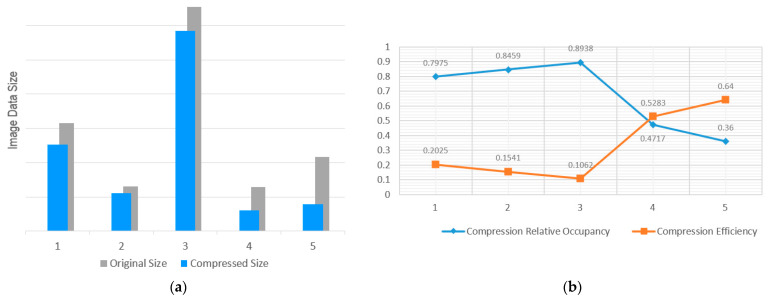
Data visualization results of the ablation experiments. (**a**) Visual results before and after image data compression. (**b**) Visual results of compression relative occupancy and compression efficiency.

The above five groups of tests are all carried out in the same environment to ensure that there is no interference from other factors. The initial state of the test environment will be restored before each test to ensure the objectivity and fairness of the test results. The test results are shown in Table 3, √ indicates that the module has been added and × indicates that the module has not been added. The verification indicators are the original size and compressed size of the image data and the compression relative occupancy rate, which is the ratio of the compressed image data size to the original size. The visualization results of the ablation experiment are shown in Figure 11. Figure 11a shows the visualization results of the image data before and after compression, and Figure 11b shows the visualization results of the image compression relative occupancy rate and compression efficiency.

According to the test results, different key modules can affect the overall image compression quality to varying degrees. Among them, the multi-scale discriminator has the greatest impact on the image compression efficiency, and other modules have relatively low image compression efficiency. Therefore, the effective implementation of high-quality image compression algorithms requires the close cooperation of all key modules. Finally, the designed high-quality image compression algorithm achieves good compression efficiency and can effectively compress a large number of images.

## 5. Conclusions

In this paper, the high-quality image compression algorithm based on unsupervised learning is studied. The traditional image compression algorithm needs preprocessing to obtain prior knowledge, which leads to low compression efficiency and the compressed image losing more details. The existing algorithms typically rely on limited datasets, which leads to poor generalization performance and unstable operation. In view of the above problems, this paper first constructs the overall network structure of the image high-quality algorithm based on unsupervised learning, proposes a content-weighted convolutional autoencoder network module to achieve image compression based on a smaller bit rate, and uses an importance map network and binary quantizer to reasonably guide the allocation of spatial bits. A generative adversarial network framework based on a multi-scale discriminator is designed to achieve end-to-end training. Through training with different weights, the accuracy distortion of each scale is minimized, thereby generating a higher quality compressed image. Experimental results show that the algorithm performance of the proposed algorithm model on more than Kodak24, Cityscapes, and Urban100 public datasets is better than that of the traditional algorithm JEPG2000 and other mainstream advanced algorithms. Its advantage is that it can better preserve the details, texture, and semantic information of the original image while achieving efficient processing of image compression tasks.

## Figures and Tables

**Table 1:** Experimental basic environment configuration.

Name	Parameter Details
CPU	Intel i7 13700F (US, CA)
GPU	Nvidia RTX 3060Ti 8 GB (US, CA)
RAM	32 GB (CN, TW)
Operating system	Windows 10 (US, WA)
Compilation tool	Pycharm 2021.3.3 (CZ, Prague)
Program framework	Tensorflow-2.11.0 (US, CA)

**Table 2:** Details of the experimental dataset.

Classification	Dataset	Number of Images	Features
Training set	ImageNet	14,197,122	Large number, high resolution, contains more irrelevant noise
Validation set	Kodak24	24	24 image compression scenes of different types and difficulties
	Cityscapes	5000	Professional images of urban environments
	Urban100	100	Contains challenging urban scenery with details in different frequency bands

**Table 3:** Test results of image batch file compression.

No.	Content-Weighted Autoencoder	Importance Map	Binary Quantizer	Multi-Scale Discriminator	Compressed Size (Bytes)	Original Size (Bytes)	Compression Relative Occupancy
1	×	√	√	√	504,509,314	632,618,579	0.7975
2	√	×	√	√	221,275,152	261,585,473	0.8459
3	√	√	×	√	1,169,481,629	1,308,437,714	0.8938
4	√	√	√	×	121,733,768	258,074,555	0.4717
5	√	√	√	√	156,688,521	435,245,891	0.3600

## Data Availability

The data are available from the authors upon reasonable request.
